# Cases Treated by Ice

**Published:** 1889-11-02

**Authors:** 


### THERAPEUTICS.

### CASES TREATED BY ICE.

In a recent number of the Philadelphia *Medical Times* we read a note of "Cases Treated by Ice to the Spine," by Dr. Kinnear, after the manner of Dr. Chapman. The cases mentioned are insomnia, herpes zroten, and spasmodic croup. Chapman's icebag, containing cracked ice, was applied to the spine in all these cases with marked success. There is no doubt that complaints such as insomnia and spasmodic croup, if not originated by disordered bowel, and if therefore purely nervous, might be benefited by ice cautiously applied to the spine. As regards ice in herpes zroten, we feel more doubtful. The author explains its action thus: "The spinal trophic and sensory centres were hyperœmic. Ice over the hyper-nourished centres expelled the excess of blood." But herpes zroten has been by some authorities regarded as due to affection of the ganglionic system, and Bärensprung held the view that the primary seat of the affection is in the spinal ganglion. If we regard herpes zroten as something other than a malady of the skin, there may be some explanation of the effects of the spinal icebag in Dr. Kinnear's cases.

